# Genomic profiling and characterization of ocular *Chlamydia trachomatis* reference strain B/HAR36

**DOI:** 10.1128/mra.00029-24

**Published:** 2024-05-03

**Authors:** Ehsan Ghasemian, Martin J. Holland

**Affiliations:** 1 Department of Clinical Research, London School of Hygiene and Tropical Medicine, London, United Kingdom; Wellesley College, Wellesley, Massachusetts, USA

**Keywords:** *Chlamydia trachomatis*, trachoma, genomics, B/Tunis864, B/HAR36

## Abstract

The human pathogen *Chlamydia trachomatis* has multiple serovariants that have distinct organotropisms. We recently revised genomic sequence data linked to ocular reference strain, B/HAR36. Now linked to its correct genomic data in the European Nucleotide Archive, we describe its genomic features.

## ANNOUNCEMENT

*Chlamydia trachomatis* (Ct), a Gram-negative intracellular bacterium, consists of 19 serovars, of which A, B, Ba, and C are responsible for trachoma, a blinding disease resulting from recurrent ocular Ct infections (1–3). Recently, we provided a European Nucleotide Archive (ENA) amendment for two Ct reference strains B/Tunis864 and B/HAR36. This amendment corrected the previous ENA accession for B/Tunis864, the genome sequence of which was incorrectly identified as B/HAR36. For this purpose, we obtained original stocks of B/HAR36 and B/Tunis864 from the laboratory of Prof. Julius Schachter at the World Health Organization Collaborating Center for Reference and Research on Trachoma and Other Chlamydial Infections, Francis I Proctor Foundation for Research in Ophthalmology, University of California, San Francisco and subjected them to Whole-Genome Sequencing (WGS) (4). Here, we provide a brief overview of the metadata and WGS statistics for both strains, focusing particularly on the genomic characteristics of strain B/HAR36 recently deposited in ENA (Table 1) (4).

**TABLE 1:** Metadata, sequence statistics, and accession numbers for the strains B/HAR36 and B/Tunis864

Ct strain	Run accession	Assembly accession	Place of isolation	Year of isolation	Isolation source	Total no. of reads	Avg. read length (bp)	Avg. quality per read	N50	Number of contigs (>1,000 bp)	Genome size (bp)	Genome GC%	No. of gene
B/HAR36	ERR12253486	GCA_963919375	Saudi Arabia	1969	Human eye	3,378,098	151	32.4	2	7	1,033,371	41.3	937
B/Tunis864	ERR12253485	GCA_963919385	Tunisia	1972	Human eye	3,083,930	151	32.3	2	32	1,044,065	41.3	947

Strains B/HAR36 and B/Tunis864 were derived from trachoma patients’ ocular samples obtained in Saudi Arabia and Tunisia, respectively, and were grown in the laboratory of Prof. Julius Schachter through the yolk-sac inoculation of embryonated eggs (5–8). DNA for WGS was extracted from original stocks using Biochain Blood and Serum kit (BioChain Institute Inc., Newark, CA, USA) following the manufacturer’s instructions. WGS of strains B/HAR36 and B/Tunis864 was conducted following the SureSelectXT (Agilent Technologies, UK) and Illumina Paired-End Sequencing Library protocols (v1.4.1 September 2012; HiSeq 2000). Read quality was assessed using the FastQC version 0.12.1 in Galaxy (Table 1) (9). Assembling the trimmed short reads in Geneious (version 2023.2.1) was accomplished using VELVET (version 1.2.10) in conjunction with VelvetOptimiser (Table 1) (10, 11). Chromosomal genes were defined using the annotated genome from Ct reference strain A/HAR13 (NC_007429) using a BLAST-like algorithm in Geneious (Table 1). For the global phylogeny, chromosomes and plasmids from 34 Ct reference strains and B/HAR36 were aligned using progressiveMauve (version 2.4.0) (12), and a tree was reconstructed using RAxML (version 8.2.11) (13). Plasticity zone (PZ) and *omp*A alignments were generated using MAFFT (version v7.490), and PhyML (version 3.3.20180621) was utilized to estimate maximum-likelihood phylogenies of aligned sequences (14–16).

**Fig 1:**
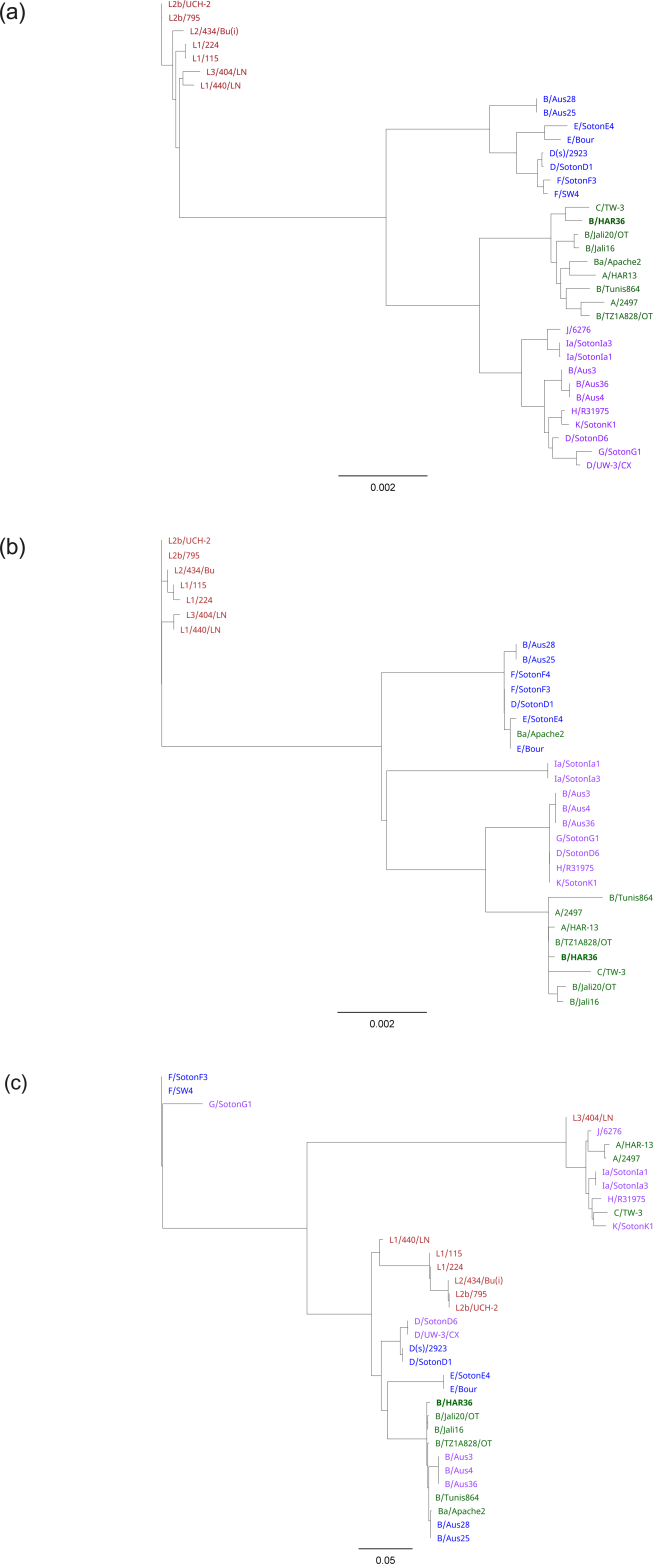
Phylogenetic reconstruction of *Chlamydia trachomatis* (Ct) chromosome (a), plasmid (b), and *omp*A gene (c). Lymphogranuloma venereum strains are represented in red, prevalent urogenital strains are represented in blue, urogenital strains are represented in violet, and ocular strains are represented in green. Ct strain B/HAR36 is highlighted in bold. The scale bar indicates evolutionary distance.

Assembling the WGS short reads resulted in contigs with complete coverage for the genomes of strains B/HAR36 and B/Tunis864. Ct global phylogeny illustrated that the chromosome of B/HAR36 clustered with C/TW-3, forming a sub-clade within the ocular clade (17) (Fig. 1a). Furthermore, the plasmid phylogeny demonstrated a close association between B/HAR36 and the cryptic plasmid of C/TW-3 (Fig. 1b). In the *omp*A maximum likelihood phylogenetic tree (Fig. 1c), B/HAR36 grouped alongside other Ct serovar B reference strains, forming a distinct cluster separate from ocular strains such as A/HAR13, A/2497, and C/TW-3 (referred to as *omp*A C-complex strains) (18).

In line with prior studies by Carlson et al. (19) and Caldwell et al. (20), we observed a genome reduction of ~10 kbp in the PZ of Ct strain B/HAR36. This reduction originates from the end of PZ, spanning 10 genes: CT_162–CT_171 partly encoding for chlamydial cytotoxin and tryptophan operons. Chlamydial PZ is known to encode niche-specific virulence determinants that dictate pathogenic diversity (21–23).

## Data Availability

Reference strains B/HAR36 and B/Tunis864 sequencing data (Accession ERR12253486 and ERR12253485, respectively) and assemblies (Accession GCA_963919375 and GCA_963919385, respectively) in the form of fastq.gz and fasta.gz files can be accessed from the European Nucleotide Archive (ENA) project accession PRJEB68374.
